# Outcomes and Predictive Factors of I-125 Plaque Therapy for Refractory Retinoblastoma

**DOI:** 10.3390/jcm14051778

**Published:** 2025-03-06

**Authors:** Yacoub A. Yousef, Farah Halawa, Mona Mohammad, Lama Al-Fahoum, Rama Soudi, Mustafa Mehyar, Reem AlJabari, Hadeel Halalsheh, Ibrahim AlNawaiseh, Imad Jaradat

**Affiliations:** 1 Department of Surgery/Ophthalmology, King Hussein Cancer Centre (KHCC), Amman 11941, Jordan; dr.f.halawa@eyehosp.com (F.H.); dm.11804@khcc.jo (M.M.); lama.anmar@hotmail.com (L.A.-F.); dr.r.alsoudi@eyehosp.com (R.S.); mmehyar@khcc.jo (M.M.); ra.11229@khcc.jo (R.A.); ia.00146@khcc.jo (I.A.); 2 Department Pediatrics Oncology, King Hussein Cancer Centre (KHCC), Amman 11941, Jordan; hadeelhalalsheh@khcc.jo; 3 Department Radiation Oncology, King Hussein Cancer Centre (KHCC), Amman 11941, Jordan

**Keywords:** retinoblastoma, I-125 plaque therapy, refractory tumors, salvage therapy, chemotherapy

## Abstract

**Objective**: This study aimed to evaluate the outcomes and predictive factors of I-125 radioactive plaque therapy for recurrent and refractory retinoblastoma (Rb) cases that failed primary systemic chemotherapy and focal therapies. **Methods**: A retrospective study of 20 eyes with intraocular Rb treated with I-125 radioactive plaque therapy (Apex dose 45 Gy) from 2013 to 2023 was conducted. Data on tumor characteristics, treatments, and outcomes were collected over a follow-up period of at least one year. **Results:** There were 11 (55%) males and 8 (40%) patients who had bilateral disease. All 20 treated eyes (100%) showed initial tumor regression, while long-term tumor control and eye salvage were achieved in 14 eyes (70%). Six eyes (30%) experienced uncontrollable tumor recurrence after a mean of 6 months (range: 3–12 months) after plaque therapy. Recurrence included main tumor activity in six eyes and additional resistant vitreous seeds in two of them. Poor predictive factors for eye salvage included Group D at diagnosis (*p* = 0.044), active vitreous seeds at the time of plaque therapy ((*p* = 0.045), tumor thickness >5.0 mm (*p* = 0.045), and tumor base dimension >12 mm (*p* = 0.023). Post-plaque complications included cataracts in seven eyes (35%), tumor hemorrhage in six eyes (30%), retinal detachment in four eyes (20%), radiation retinopathy in three eyes (15%), and neovascular glaucoma in one eye (5%). Five (83%) of those with tumor hemorrhage had plaque surgery performed within less than 6 months of the last cycle of systemic chemotherapy. At a mean follow-up of 36 months (range: 12–96 months), five eyes (25%) were enucleated, and high-risk pathological features were identified in three eyes, including post-laminar optic nerve infiltration (one eye) and massive choroidal invasion (two eyes). All patients were alive and free of metastasis except one patient (5%) whose parents refused enucleation and came back with extra-scleral extension and bone marrow metastasis and eventually passed away. **Conclusions**: I-125 radioactive plaque therapy is a valuable salvage treatment for recurrent and refractory retinoblastoma, achieving tumor control and eye salvage in 70% of cases with an acceptable safety profile. However, the observed recurrence rate (30%) at an apex dose of 45 Gy suggests a need for dose optimization and individualized treatment strategies. Identifying high-risk features, such as Group D disease, active vitreous seeds, and larger tumors, is crucial for patient selection and outcome prediction. Future research should explore alternative dosing strategies, combination therapies, and improved predictive models to enhance long-term tumor control while minimizing complications.

## 1. Introduction 

Retinoblastoma (Rb) is the most common primary intraocular malignancy in children, with an incidence of 1 in 15–20 thousand live births [1,2]. Multiple treatment modalities have been utilized for RB, including systemic chemotherapy (IVC), intra-arterial chemotherapy (IAC), intravitreal chemotherapy (IViC), cryotherapy, laser therapy, plaque radiotherapy, and enucleation. And each treatment modality has specific benefits and risks [3,4,5,6,7,8,9,10].

Intravenous chemotherapy is used most widely as the primary treatment for retinoblastoma, in combination with laser photocoagulation, cryotherapy, and thermochemotherapy [11,12,13,14]. However, plaque brachytherapy [15], which is used for thick tumors that cannot be cured by laser therapy and cryotherapy, is still used as an approach in an attempt to salvage the eye, mainly in situations where globe salvage is desired, like, for example, when the opposite eye is enucleated. The advantage of plaque therapy is that localized radiation to the eye will not increase the chance of a second malignancy, which is a major disadvantage for external beam radiation therapy (EBRT) [16,17,18]. Focal treatment such as plaque radiotherapy has been utilized either as a primary or as a salvage therapy following systemic IVC or IAC for focal new or recurrent tumors, and eye salvage rates are reported to range from 55% to 79% [15,19,20]. 

Plaque therapy has been employed at KHCC since 2008 for the treatment of retinoblastoma and uveal melanoma [21,22]. Herein, we evaluated the outcome and the factors affecting the outcome of using Iodine-125 (I-125) radioactive plaque therapy in the management of resistant and recurrent intraocular retinoblastoma cases that failed treatment by systemic chemotherapy combined with focal laser therapy and/or cryotherapy. 

## 2. Patients and Methods 

This is a retrospective study of 20 eyes from 20 patients who had a clinical diagnosis of intraocular Rb and were treated with I-125 radioactive plaque therapy. The study period spans from January 2013 to December 2023. The Institutional Review Board at KHCC approved the study. 

Each patient underwent a comprehensive ophthalmic examination performed under general anesthesia that included various assessments such as indirect ophthalmoscopy, ocular ultrasound B-scan, and brain magnetic resonance image (MRI) scan as needed. Fundus photos were taken by Retcam II (Clarity Medical Systems, Pleasanton, California) for documentation. Selection required access to patients’ medical, radiological, and pathological reports and fundus Ret-Cam images. Data collected included the patient’s age, gender, family history, laterality, age at diagnosis, disease stage, treatment modalities, follow-up, eye salvage, metastasis, and survival. 

### 2.1. Inclusion and Exclusion Criteria

Only the eyes with the clinical diagnosis of Rb that were treated at one stage by I-125 radioactive plaque therapy and followed for at least 1 year after plaque therapy were included. Eyes that were followed for less than one year were excluded. The radioactive plaque was offered only to patients with intraocular retinoblastoma with an absence of anterior segment invasion, absence of extra scleral or optic nerve invasion, and tumor thickness less than 10 mm. 

### 2.2. Clinical Characteristics and Definitions

Tumors were staged at presentation according to IIRC [23] and 8th edition TNM staging systems [24]. At the time of plaque therapy, tumors were divided into two groups based on the presence or absence of active vitreous seeds. Recurrent tumors were defined as tumor reactivation after 6 months of absence of any clinical signs of activity, and refractory tumors were defined as continuously active tumors even after completion of systemic chemotherapy. Failure of treatment after radioactive plaque therapy was defined as any uncontrollable tumor progression or relapse after plaque therapy that ended with a team decision of enucleation. Eye salvage was defined as tumor control and avoidance of enucleation and external beam radiation therapy. Visual acuity for children was evaluated using an LEA chart when possible.

### 2.3. Treatment Methods

We performed a combination regimen of chemotherapy that consisted of CVE (carboplatin, vincristine, and etoposide). Each CVE cycle was repeated every 4 weeks for a total of 6 to 8 cycles according to the patient’s condition and tumor status. Ocular oncology follow-up was provided as examination under general anesthesia before each cycle of chemotherapy and every 4 to 8 weeks thereafter. Fundus photos were taken using a RetCam II (Clarity Medical System, Pleasanton, CA, USA), and focal therapy was provided using thermotherapy and/or cryotherapy. 

Radioactive plaque therapy was administered in a consistent I-125 COMS plaque, with an apex dose of 45 Gy placed under general anesthesia for 2 days before removal. Active vitreous seeds were treated by IViC after the plaque therapy if clinical activity was shown. 

### 2.4. Statistical Analysis

Statistical analysis of tumor control and eye salvage were correlated to demographics and tumor features. The *p* value was measured to test the predictive power of each factor using the exact Fisher test, and a *p* value less than 0.05 was considered significant.

## 3. Results 

### 3.1. Demographics and Clinical Features 

Twenty eyes with intraocular Rb from 20 patients were studied. The mean age at diagnosis was 24 months (median 12 months, range; 3–72 months). There were eleven (55%) males and nine (45%) females. Eight (40%) had bilateral disease, and three (15%) had a positive family history of Rb. All of them were treated with I-125 radioactive plaque therapy. The demographics are shown in Table 1.

### 3.2. Tumor and Plaque Features

According to the international classification of intraocular retinoblastoma (IIRC), most of the eyes (60%) were Group D, and all (100) were T2 according to the 8th edition TNM staging (Table 2). The indication for plaque therapy was recurrent tumors in eight (40%) eyes and refractory tumors in twelve (60%) eyes. The details of tumor characteristics and plaque features are summarized in Table 2. All eyes were treated with I-125 plaque, the mean apical dose was 43.5 Gy (range 41–46 Gy), the scleral dose ranged from 150–280 Gy, and the duration of the plaque inside the eye was 44–48 hrs. All plaques were regular COMS plaques except four (20%) that were notched plaques. 

### 3.3. Treatment Outcomes and Complications

All (100%) of the 20 eyes treated showed initial tumor regression, which was maintained with non-recurrence with eye salvage in 14 (70%) eyes. Six (30%) eyes showed uncontrollable tumor recurrence after a mean of 6 months (range 3–12 months). Tumor recurrence was seen in six eyes; four had progressive main tumor growth, and two had marginal recurrence. The two eyes with marginal recurrence had received a notched plaque that created geographic miss. Two of the four eyes with main tumor progression had concomitant resistant vitreous as well. 

**Figure 1:**
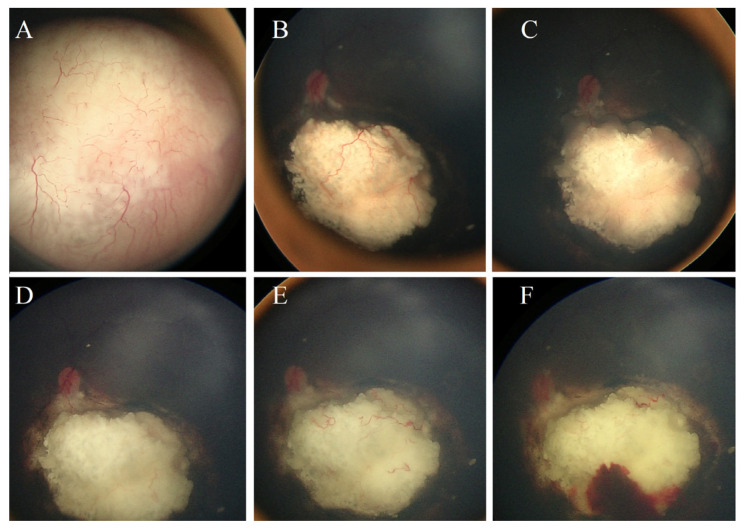
Clinical course of a 2-year-old girl with left unilateral retinoblastoma (Group D). (**A**) Initial presentation showing the tumor before treatment. (**B**) Complete tumor regression following six cycles of CVE chemotherapy and focal consolidation therapy using Transpupillary Thermotherapy (TTT). (**C**) Significant tumor recurrence observed three months after the last chemotherapy cycle. (**D**) Complete tumor regression two months after I-125 radioactive plaque therapy. (**E**) Sustained tumor regression for an additional two months. (**F**) Tumor-associated hemorrhage detected six months post-plaque therapy, which resolved gradually without intervention over six months. At two years post-plaque therapy, no tumor activity was detected.

**Figure 2:**
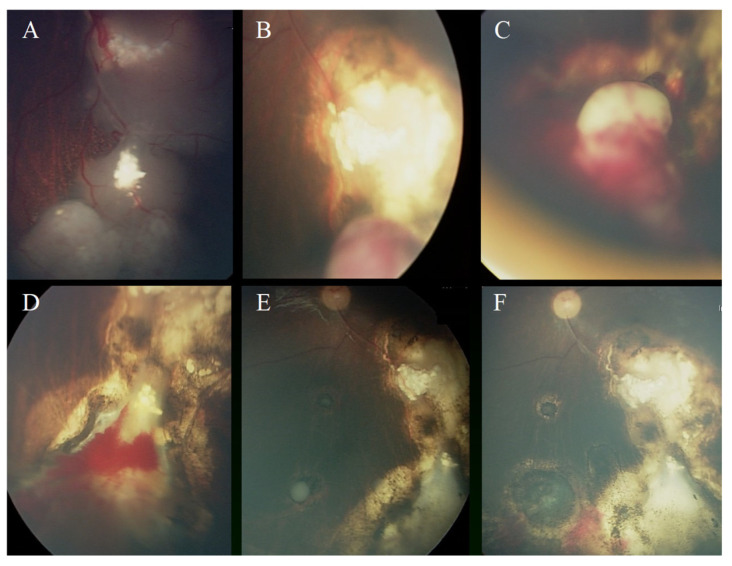
A 2-year-old single-eye patient was referred after 6 cycles of chemotherapy (VCR/Carbo/VP-16) and 2 cycles of Topotecan/VCR. (**A**) Active tumor treated with massive TTT and cryotherapy. (**B**,**C**) Partial response but residual active tumor unresponsive to cryotherapy. (**D**) Fundus photo 3 months after I-125 plaque therapy shows excellent tumor response. (**E**) Recurrent vitreous seeds 3 months after plaque therapy, which was treated successfully with intravitreal melphalan. (**F**) After 3 years of follow-up, no tumor activity. Dense cataract treated with IOL implantation; vision 20/30.

The significant poor predictive factors for eye salvage by radioactive plaque therapy were as follows: Group D at diagnosis, concomitant active vitreous seeds at the time of plaque therapy, tumor thickness more than 5.0 mm, and tumor base dimension more than 12 mm (Table 2). TNM stage, tumor location, indication for therapy (recurrent vs. refractory), and type of plaque therapy (COMS vs. notched) were not significant predictive factors Table 2. The post-plaque therapy complications included cataracts in seven (35%) eyes, tumor hemorrhage in six (30%) eyes, retinal detachment in four (20%) eyes, radiation retinopathy in three (15%) eyes, and neovascular glaucoma in one (5%) eye (Figure 1 and Figure 2). 

Of interest, five of six eyes (83%) that showed tumor hemorrhage after plaque therapy had the plaque therapy within less than 6 months of the last cycle of chemotherapy. In all of them, the hemorrhage was localized around the tumor and regressed gradually over 3–6 months, and no residual tumor was detected underneath any of them. 

All the seven eyes that had cataracts had the treated tumors located anterior to the equator. After plaque therapy, all five eyes with active vitreous seeds received melphalan IViC (mean, median 3 injections, and range 3–6 injections); two showed complete regression, while three had resistant vitreous seeds.

At a median follow-up of 36 months (range 12–96 months), 14 (70%) eyes were salvaged, and 6 (30%) eyes had unsalvageable tumor activity. Five (25%) eyes were eventually enucleated, and high-risk pathologic features were detected in three eyes (one had post-laminar optic nerve infiltration, and two had massive choroidal invasion). Parents for one (5%) patient (who was a single-eye patient) refused enucleation and was lost for follow-up and came back 12 months after the plaque therapy with extra-scleral extension and bone marrow metastasis; this patient eventually passed away. 

## 4. Discussion

Despite significant advancements in the management of intraocular Rb, including IAC and IViC, tumor recurrence remains a concern during the early follow-up period after treatment. Recurrence may present as solid tumors, vitreous seeding, sub-retinal seeding, aqueous seeding, or the emergence of new retinal tumors [25,26,27,28]. Focal treatments, such as plaque radiotherapy, have been used as both primary and salvage therapies for new or recurrent tumors, particularly following systemic IVC or IAC, and also after the failure of intravitreal or periocular chemotherapy [15,29,30,31,32,33,34,35,36]. In centers without access to plaque therapy, alternative approaches like additional cycles of systemic IVC or IAC, combined with focal treatments, may be employed. In our study, where we analyzed 20 consecutive eyes with Rb, I-125 radioactive plaque therapy proved to be an effective treatment for recurrent or refractory intraocular Rb, yielding positive results in terms of both tumor regression and eye salvage. The radioactive plaque therapy induced tumor regression in all treated eyes initially, with 70% of eyes maintaining tumor control over a median follow-up period of 36 months. However, a significant challenge was the recurrence of tumor activity in 30% of the eyes, leading to the eventual enucleation. Plaque therapy was associated with certain complications, such as cataracts, retinal hemorrhages, and retinal detachment, aligning with expectations for radiation-induced sequelae. In enucleated eyes, pathological examination revealed high-risk features in 25% of cases that may cause metastasis if not enucleated in the adequate time. 

For tumors located anterior or posterior to the equator with a height greater than 3 mm, radioactive plaque brachytherapy using 106Ru or 125I isotopes remains the treatment of choice. The reported local tumor control rates for these isotopes range from 55% to 90% [20,29,35]. Shields et al. [15] documented success in their large cohort of 208 eyes with retinoblastoma, reporting a Kaplan–Meier estimate of tumor control of 83% at 1 year and 79% at 5 years, closely mirroring our own 70% success rate. This consistency across studies supports the robustness of plaque radiotherapy as a possible second-line treatment for controlling intraocular retinoblastoma after the failure of systemic chemotherapy combined with focal consolidation therapy. Later on, Shields et al. analyzed 84 eyes with Rb that had solid recurrence after CRD and were treated with custom-designed I125 plaque radiotherapy [14]. They excluded eyes with vitreous or sub-retinal seed recurrence, and they achieved tumor control in 95% of eyes by 5-year follow-up. This aligns with our results that all (100%) tumors showed initial regression. Their higher final tumor control rate can be explained by the fact they excluded eyes with sub-retinal or vitreous seeding, while we included them in our cohort. We found that Group D eyes were considered a poor prognostic factor for tumor control because they were associated with massive vitreous and sub-retinal seeding at diagnosis. Furthermore, active vitreous seeds at the time of treatment by I-125 radioactive plaque therapy were shown to be a significantly poor prognostic factor for eye salvage, even in the presence of IViC. Yarovoy et al. [20] focused on experience using 106Ru plaques as a salvage treatment for new or recurrent retinal tumors post-systemic IVC, like in our cohort, and showed a 55% eye salvage rate. In our cohort, we used the I-125 isotope, and the eye salvage rate was 70%. In both cases, the apex dose was around 45 Gy. In the study by Palkonda et al., the initial tumor control rate was 82% (36 eyes) in the immediate treatment period, while the long-term tumor control rate dropped to 56% (25 eyes). This was similar to our study, where the initial response was seen in all cases, but the eye salvage dropped to 70%. In our series, tumor control was maintained over a mean follow-up of 3 years, although about one-third of tumors recurred after initial control. The mean interval from plaque therapy to recurrence in the area treated with plaque was 6 months, highlighting the importance of follow-up during the first 6 months after treatment. This indicates that Rb may be resistant to the apex dose of 45 Gy eyes treated with I-125 and 106Ru; therefore, the apex dose may need to be adjusted to a higher dose, which unfortunately may be reflected badly in side effects.

The factors predictive of local tumor recurrence after plaque therapy include older age, absence of previous focal laser therapy, advanced tumors larger than 5 disc diameters, vitreous seeds, partial or complete tumor regression, and prior external beam radiotherapy [14,15,16,32,33,36]. These factors emphasize the need for long-term follow-up to detect recurrence early. In our series, massive tumor seeds at diagnosis (Group D), active vitreous seeds at the time of plaque therapy, tumor base dimension of more than 12 mm, and thickness of more than 5 mm were considered as poor predictive factors for eye salvage. 

One of the poor predictive factors for tumor control in our study was the base dimension of the tumor, with eyes that have a higher base diameter than 12 mm being less salvageable. Tumors with a base dimension of less than or equal to 12 mm had a 100% salvage rate compared to a 50% salvage rate in tumors greater than 12 mm, with a *p* value of 0.023. This finding supports the idea that smaller tumors are more responsive to radiotherapy and that larger tumors are more challenging to control with localized radiation due to uneven dose distribution and more aggressive biological behavior. This agrees with the studies that were conducted to describe the clinical presentation and treatment outcomes of patients undergoing Ruthenium-106 (106Ru) plaque brachytherapy as a salvage treatment for retinoblastoma (RB) following intravenous chemotherapy (IVC) [20]. Furthermore, even though the type of plaque therapy (COMS vs. Notched) was not considered as a statistically significant parameter to predict outcome in our study, we found that 75% of eyes treated by COMS plaque were salvaged compared to 50% of eyes treated by notched plaque. This is attributed to the fact that COMS plaques offer uniform radiation distribution over the tumor and surrounding tissues, whereas notched plaques may cause irregular radiation distribution, leading to insufficient radiation to certain areas of the tumor [14,15,16].

Long-term radiation-related complications, such as retinopathy, optic neuropathy, and cataracts, have been reported following the use of 106Ru [20]. In our study using I125, a mean dose of 45 Gy was delivered, resulting in a tumor control and globe salvage rate of 70%. The most common complications observed in our series were cataracts (35%), tumor hemorrhage (30%), retinal detachment (20%), and radiation retinopathy (15%). Other studies have shown varied results, with slightly higher maculopathy rates when plaque therapy is used as primary treatment compared to salvage therapy [14,15,16,20,28,31,32,33,34]. This difference may be attributed to the larger tumor size and cumulative dose in the primary treatment group. The lower rate of radiation retinopathy and higher rate of cataracts may be due to the location of the tumor. In our study, in all seven eyes that had cataracts, the treated tumors were located anterior to the equator. Of interest, 83% of eyes that showed tumor hemorrhage after plaque therapy had plaque therapy within less than 6 months of the last cycle of chemotherapy. This may indicate that plaque therapy is better to be post-ponded for at least 6 months after the last cycle of systemic chemotherapy to avoid severe tumor-related hemorrhages.

This study examined the outcome and the predictive factors for tumor control by I-125 radioactive plaque therapy for retinoblastoma in eyes that were resistant to chemotherapy and focal therapy. Although our results are important, this study has some limitations, including the small sample size and retrospective design. Therefore, a larger and more comprehensive multicenter study should be performed to better assess the efficacy and tumor-specific factors expected from radiation therapy, justify the possible associated risks, and evaluate the real radiation dose that is needed to eradicate Rb.

In conclusion, I-125 radioactive plaque therapy proves to be a valuable and effective salvage treatment for recurrent and refractory retinoblastoma cases that have failed systemic chemotherapy and focal therapies. The therapy achieved initial tumor regression in all treated eyes, with a 70% success rate in long-term tumor control and eye salvage. However, the persistence of tumor activity in 30% of cases highlights the need for ongoing vigilance and alternative management options for resistant tumors or the need to reconsider a higher dose than the conventional 45 Gy apex dose to avoid tumor resistance. While complications such as cataracts, retinal hemorrhage, and retinal detachment were observed, the overall safety profile remains manageable; however, radioactive plaque therapy should not be concomitant with systemic chemotherapy to avoid hemorrhages. Predictive factors such as tumor thickness, base dimension, and the presence of vitreous seeds were found to influence the likelihood of eye salvage, emphasizing the importance of early and appropriate intervention. Our findings contribute to the growing body of evidence supporting I-125 plaque therapy as a reliable second-line treatment in the management of intraocular Rb. 

## Figures and Tables

**Table 1:** Demographics and clinical features for 20 eyes for 20 patients with intraocular retinoblastoma treated By I-125 radioactive plaque therapy.

Feature		Number	Percentage	Salvage	*%*	*p* Value *
		20		14	70%	
Age	Median, Mean, Range		12 months, 24 months, range 3–72 months			
	Less than 12 months	7	35%	5	71%	0.77
	12–24 months	3	15%	2	67%	
	More than 24 months	10	50%	7	70%	
Gender	Male	11	55%	8	72%	0.77
	Female	9	45%	6	66%	
Family history	Positive	3	15%	2	66%	0.89
	Negative	17	85%	12	71%	
Laterality	Unilateral	12	60%	8	67%	0.69
	Bilateral	8	40%	6	75%	
Side	Right	14	70%	10	71%	0.83
	Left	6	30%	4	67%	

* p value less than 0.05 was considered significant.

**Table 2:** Tumor characteristics, plaque features, and eye salvage rates for 20 eyes with intraocular retinoblastoma treated by I-125 radioactive plaque therapy.

Feature		Number	Percentage	Salvage	*%*	*p* Value
IIRC stage	C	7	40%	7	88%	0.044
	D	13	60%	7	58%	
TNM stage	cT2a	2	10%	2	100%	0.32
	cT2b	18	90%	12	67%	
Thickness	Less than or equal to 5 mm	13	62%	11	85%	0.045
	More than 5 mm	7	35%	3	43%	
Base dimension	Less than or equal to 12 mm (plaque size 16)	8	40%	8	100%	0.023
	More than 12 mm (plaque size 18)	12	60%	6	50%	
Distance to macula	Macular	7	35%	5	71%	0.66
	Extramacular	13	65%	9	69%	
Vitreous seeds *	Yes	5	25%	2	40%	0.045
	No	15	75%	12	80%	
Indication	Recurrent	8	40%	5	63%	0.27
	Refractory	12	60%	9	75%	
Plaque type	COMS	16	80%	12	75%	0.34
	Notched	4	20%	2	50%	

* Active vitreous seeds at the time of radioactive plaque therapy.

## Data Availability

The research data is available upon reasonable request.
